# Sleep experiences during different lifetime periods and in vivo Alzheimer pathologies

**DOI:** 10.1186/s13195-019-0536-6

**Published:** 2019-09-12

**Authors:** Young Min Choe, Min Soo Byun, Dahyun Yi, Jun Ho Lee, So Yeon Jeon, Bo Kyung Sohn, Yu Kyeong Kim, Seong A Shin, Chul-Ho Sohn, Yu Jin Lee, Dong Young Lee, for the KBASE Research Group

**Affiliations:** 1 Department of Neuropsychiatry, Hallym University Dongtan Sacred Heart Hospital, Hwaseong, Republic of Korea; 2 Institute of Human Behavioral Medicine, Medical Research Center, Seoul National University, Seoul, Republic of Korea; 3 Department of Neuropsychiatry, Seoul National University Hospital, Seoul, Republic of Korea; 4 Department of Neuropsychiatry, Chungnam National University Hospital, Daejeon, Republic of Korea; 5 Department of Psychiatry, Sanggye Paik Hospital, Inje University College of Medicine, Seoul, Republic of Korea; 6 Department of Nuclear Medicine, SMG-SNU Boramae Medical Center, Seoul, Republic of Korea; 7 Department of Radiology, Seoul National University Hospital, Seoul, Republic of Korea; 8 Center for Sleep and Chronobiology, Seoul National University Hospital, Seoul, Republic of Korea; 9 Department of Psychiatry, Seoul National University College of Medicine, Seoul, Republic of Korea

**Keywords:** Midlife sleep, Preclinical Alzheimer’s disease, Cerebral amyloid, Neurodegeneration

## Abstract

**Background:**

Very little is known for the direction or causality of the relationship between lifetime sleep experiences and in vivo Alzheimer’s disease (AD) pathologies. This study aimed to examine the relationship between sleep experiences during the young adulthood, midlife, and late-life periods and in vivo cerebral beta-amyloid (Aβ) deposition and AD signature regional neurodegeneration in cognitively normal (CN) old adults.

**Methods:**

This study included 202 CN old adults who participated in the Korean Brain Aging Study for the Early Diagnosis and Prediction of Alzheimer’s Disease (KBASE) study. All participants underwent a comprehensive clinical assessment, [^11^C] Pittsburgh Compound B positron emission tomography (PET), [^18^F] Fluorodeoxyglucose-PET, and magnetic resonance imaging. The quality and duration of sleep were assessed for the following age periods: 20–30s, 40–50s, and the most recent month. All analyses were adjusted for age, gender, education, apolipoprotein E ε4 status, vascular risk score, Hamilton Depression Rating Scale score, and use of sleep medication.

**Results:**

Bad sleep quality and short sleep duration during midlife were significantly associated with increased Aβ deposition and AD signature regional hypometabolism, respectively. Although current bad sleep quality appeared to be associated with increased Aβ accumulation, this association disappeared after controlling for the effects of midlife sleep quality. Neither the quality nor duration of sleep during young adulthood was related to Aβ burden or neurodegeneration.

**Conclusions:**

Bad sleep quality during midlife increases pathological Aβ deposition in the brain, while short sleep duration during the same period accelerates regional hypometabolism.

**Electronic supplementary material:**

The online version of this article (10.1186/s13195-019-0536-6) contains supplementary material, which is available to authorized users.

## Background

A number of studies have shown that disturbed sleep and Alzheimer’s disease (AD) dementia are closely related [1–3]. Further, experimental evidence suggests that the relationship between poor sleep and beta-amyloid (Aβ) accumulation, which is a pathological hallmark of AD, is bidirectional [4]. For example, Aβ aggregation disrupts the sleep-wake cycle as well as the diurnal fluctuation of Aβ in mice [5], and conversely, acute sleep deprivation increases brain Aβ levels and chronic sleep restriction increased Aβ plaque formation in transgenic mice [6]. It has also been suggested that AD pathophysiology impairs the sleep-regulating system including hypocretin and melatonin regulation, and reversely, hypocretin and melatonin may play an important role in modulating AD pathophysiology [7].

Several clinical studies based on amyloid positron emission tomography (PET) imaging have revealed that poorer self-reported sleep quality is associated with a greater in vivo Aβ burden in the human brain [8–11], while the association between self-reported sleep duration and Aβ deposition is controversial [8, 10–12]. However, all studies that have investigated this relationship had cross-sectional designs and merely correlated current sleep experience with Aβ; thus, they could not provide any clue on the direction or causality of the relationship. Two translational human studies partly demonstrated the causal relationship; one night of sleep deprivation increased cerebrospinal fluid (CSF) Aβ42 [13], and slow-wave activity disruption increased CSF Aβ40 [14]. However, they only tested the acute effect of disrupted sleep, which may differ from the lifelong effects of poor sleep.

To overcome these limitations, we assessed sleep experiences, including sleep quality and sleep duration, of cognitively normal (CN) old adults during three periods of life: young adulthood, midlife, and late-life. Rather than merely assessing the cross-sectional association between current sleep and pathology, this information was used to examine the influences of each lifetime sleep experience on in vivo cerebral Aβ deposition. In addition, the associations of lifetime sleep experiences with AD signature regional neurodegeneration, as measured using cerebral glucose metabolism and cortical thickness, were evaluated. Only limited information is available regarding this association [9, 15].

## Methods

### Participants

The present study was part of the Korean Brain Aging Study for the Early Diagnosis and Prediction of Alzheimer’s Disease (KBASE) [16], which is an ongoing prospective cohort investigation, and included a total of 202 CN elderly individuals from the KBASE cohort. The inclusion criteria were being aged from 60 to 90 years old, a Clinical Dementia Rating score of 0, and no diagnosis of mild cognitive impairment or dementia. The exclusion criteria were as follows: the presence of any serious medical, psychiatric, or neurological disorder that could affect mental functioning; major sleep disorder other than insomnia disorder (e.g., rapid eye movement sleep behavior disorder, obstructive sleep apnea, and/or restless legs syndrome); the presence of severe communication problem that would make a clinical examination or brain scan difficult; contraindications for a magnetic resonance imaging (MRI) scan; the absence of a reliable informant; illiteracy; and/or participation in another clinical trial of sleep treatment.

### Clinical assessment

All participants underwent a standardized clinical evaluation conducted by trained psychiatrists which was based on the KBASE clinical assessment protocol, which incorporates the Korean version of the Consortium to Establish a Registry for Alzheimer’s Disease Assessment Packet (CERAD-K) [17]. The KBASE neuropsychological assessment protocol that incorporates the CERAD neuropsychological battery [18] was also given to all participants by trained neuropsychologists. The presence of six vascular risk factors, including hypertension, diabetes, dyslipidemia, transient ischemic attack, stroke, and coronary artery disease, was systematically evaluated; a vascular risk score (VRS) was calculated as the total number of the vascular risk factors and reported as a percentage [19]. Depressive symptoms were measured using the Hamilton Depression Rating Scale (HDRS) [20].

### Assessment of sleep experiences

All participants were systematically interviewed by trained nurses using a structured interview form to determine past average sleep duration and subjective sleep quality during the following lifetime periods: 20–39 (young adulthood) and 40–59 (midlife) years. The participants also completed the Pittsburgh Sleep Quality Index (PSQI) [21]; of the 18 PSQI items, items assessing sleep duration (item #4) and subjective sleep quality (item #18) were used for the evaluation of current (i.e., late-life) sleep experiences. For all past (i.e., young adulthood and midlife) and current sleep experiences, sleep duration was reported as the mean hour of sleep and subjective sleep quality was rated on a 4-point scale (“very good,” “fairly good,” “fairly bad,” and “very bad”). Information about current and past sleep disorders and the current use of sleep medication was also obtained through the structured interview and a review of medical records by trained nurses.

### Neuroimaging evaluation

#### Aβ deposition

All participants underwent simultaneous three-dimensional (3D) [^11^C] Pittsburgh compound B (PiB) PET and 3D T1-weighted MRI scans using a 3.0-T Biograph mMR (PET-MR) scanner (Siemens; Washington DC, USA); the details of the PiB-PET and MRI acquisition and preprocessing have been described in a previous report from our research group [22]. The automated anatomical labeling algorithm [23] and a region-combining method [24] were applied to determine the region of interests (ROIs) to characterize ^11^C-PiB retention levels in the frontal, lateral parietal, posterior cingulate-precuneus, and lateral temporal regions of the brain. The standardized uptake value ratio (SUVR) for each ROI was calculated by dividing the mean value for all voxels within each ROI by the mean cerebellar uptake value [25]. Participants were classified as Aβ positive if the SUVR was > 1.4 in at least one of the four ROIs or as Aβ negative if the SUVR each of the four ROIs was ≤ 1.4 [24, 26]. A global Aβ retention value was generated by dividing the mean value for all voxels of the global cortical ROI by the mean cerebellar uptake value in the same image [24, 25, 27].

#### AD signature region neurodegeneration

Participants also underwent 3D [^18^F] fluorodeoxyglucose (FDG) PET imaging using the same PET-MR machine described above. The details of the FDG-PET image acquisition and preprocessing have been described in a previous report from our research group [22]. Voxel-weighted mean SUVRs were extracted from AD signature FDG ROIs known to be sensitive to the metabolic changes associated with AD [27, 28], including the angular gyri, posterior cingulate cortex, and inferior temporal gyri. AD signature region cerebral glucose metabolism (AD-CM) was defined as the voxel-weighted mean SUVR of the AD signature FDG ROIs. AD signature region cortical thickness (AD-CT) on the MRI scans was defined as the mean cortical thickness values obtained from AD signature regions, including the entorhinal, inferior temporal, and middle temporal cortices and fusiform gyrus, as previously described [27]. AD signature neurodegeneration (AD-ND) positivity was defined as an AD-CM < 1.386 and/or an AD-CT < 2.666 mm, as described in previous studies [27–29].

### Apolipoprotein E genotyping

Genomic DNA was extracted from whole blood samples, and apolipoprotein E (APOE) genotyping was performed as previously described [30]. APOE ε4 (APOE4) positivity was coded if at least one ε4 allele was present.

### Statistical analyses

For the present analyses, self-reported sleep quality was originally recorded using a 4-point scale but then was transformed into a dichotomous variable (“good” or “bad”) because only a small number of participants reported “very bad” sleep quality. Duration of sleep was treated as a continuous variable. Multiple logistic regression analyses were performed to examine the association between sleep quality (or sleep duration) during each lifetime period and the rate of Aβ (or AD-ND) positivity after controlling for age, gender, education, APOE4, VRS, current use of sleeping pills, and HDRS score. In addition, multiple linear regression analyses that treated global Aβ deposition and neurodegeneration biomarkers as dependent variables were conducted after controlling for the same covariates used in the logistic regression analyses. Global Aβ deposition and AD-CT were natural log-transformed to achieve a normal distribution. In the event that a sleep variable was significantly related to an imaging variable, the moderating effects of APOE4 were further explored by also including a sleep variable × APOE4 interaction term as an independent variable after controlling for the same covariates. All statistical analyses were conducted using IBM SPSS Statistics 21, and *p* values < 0.05 were considered to indicate statistical significance.

## Results

### Demographics and clinical characteristics

The characteristics of the study participants are presented in Table 1. After the sleep quality based on the 4-point scale was transformed into a dichotomous variable (“good” or “bad”), the proportions of bad sleep were 6.4%, 10.4%, and 11.9% for the young adulthood, midlife, and current lifetime periods, respectively. Although the percentage of bad sleepers increased with age, the differences were not statistically significant (chi-square value = 2.496, *p* = 0.287). The mean durations of sleep were 6.83, 6.65, and 6.42 h for each lifetime period; these differences were significant according to an analysis of variance (ANOVA; *F* = 6.048, df = 2, *p* = 0.003).

**Table 1:** Demographics and clinical characteristics of study subjects (*n* = 202)

Age, years	71.07 (6.74)
Female	102 (50.5%)
Education level, years	11.51 (4.77)
APOE4 carrier	37 (18.3%)
Vascular risk score, %	17.49 (15.84)
HDRS	0.74 (1.51)
Current use of any sleep medication	6 (3.0%)
Bad sleep quality (bad + very bad)	
Young adulthood	13 (6.4%)
Midlife	21 (10.4%)
Current	24 (11.9%)
Sleep duration, h	
Young adulthood	6.83 (1.11)
Midlife	6.65 (1.18)
Current	6.42 (1.31)
Aβ positive	33 (16.3%)
Global Aβ deposition	1.20 (0.25)
AD-ND positive	112 (55.4%)
AD-CM	1.41 (0.12)
AD-CT, mm	2.84 (0.18)

Data are presented as mean (standard deviation) or number (%)

*APOE4* apolipoprotein E ε4, *HDRS* Hamilton Depression Rating Score, *Aβ* beta-amyloid, *AD-ND* Alzheimer’s disease signature region neurodegeneration, *AD-CM* Alzheimer disease signature region cerebral glucose metabolism, *AD-CT* Alzheimer’s disease signature region cortical thickness

### Associations between sleep experiences and Aβ deposition

Multiple logistic regression analyses revealed that midlife and current sleep qualities were significantly associated with Aβ positivity whereas young adulthood sleep quality was not (Table 2). The bad sleep qualities in the midlife and current periods were associated with 4.636-fold and 4.194-fold increases in the probability of Aβ positivity, respectively. Additional analyses including the sleep quality × APOE4 interaction term revealed that the interaction term was not significant for either the midlife or current lifetime period. Sleep duration in any lifetime periods was not related to Aβ positivity. As shown in Table 2, the multiple linear regression analyses revealed that midlife sleep quality had a significant association with global Aβ deposition. By contrast, there were no significant relationships between another lifetime sleep quality and global Aβ deposition or between sleep duration in any lifetime periods and Aβ deposition.

**Table 2:** Association between lifetime sleep experience and Aβ positivity (or global Aβ deposition)

	Sleep quality				Sleep duration			
	Logistic regression		Linear regression		Logistic regression		Linear regression	
	aOR (95% CI)^a^	*p*	Beta^b^	*p*	aOR (95% CI)	*p*	Beta	*p*
Young adulthood	1.864 (0.434–8.016)	0.403	0.058	0.401	1.373 (0.913–2.063)	0.128	0.099	0.148
Midlife	4.636 (1.448–14.844)	0.010	0.174	0.012	1.170 (0.804–1.703)	0.412	0.056	0.419
Current	4.194 (1.156–15.218)	0.029	0.134	0.091	0.905 (0.665–1.233)	0.528	− 0.028	0.685

Both logistic and linear regression analyses were adjusted for age, gender, education, APOE4 status, vascular risk score, use of sleep pill, and Hamilton Depression Rating Score. Global Aβ deposition was natural log-transformed to achieve normal distribution

*Aβ* beta-amyloid, *aOR* adjusted odds ratio, *CI* confidence interval, *APOE4* apolipoprotein E ε4

^a^Good sleep quality was taken as a reference, so aOR represents the likelihood of being Aβ positive for bad sleep quality compared with good one

^b^Positive beta value means that worse sleep quality is associated with increased Aβ deposition

Table 3 shows the results from the multiple logistic regression analysis that included both midlife and current sleep qualities as independent variables and Aβ positivity as a dependent variable. Midlife sleep quality was significantly associated with Aβ positivity whereas late-life sleep quality was not.

**Table 3:** Multiple logistic regression analysis with midlife and current sleep qualities as independent variables and Aβ positivity as a dependent variable

Covariates	aOR (95% CI)	*p* value
Age	1.112 (1.041–1.188)	0.002
Gender	1.043 (0.424–2.563)	0.927
Education	1.056 (0.954–1.168)	0.292
APOE4 status	3.314 (1.297–8.467)	0.012
Vascular risk score	0.987 (0.960–1.015)	0.357
Use of sleep pill	4.606 (0.715–29.659)	0.108
HDRS	0.833 (0.572–1.212)	0.340
Midlife sleep quality	3.796 (1.117–12.895)^a^	0.033
Current sleep quality	3.183 (0.793–12.779)^a^	0.103

*Aβ* beta-amyloid, *aOR* adjusted odds ratio, *CI* confidence interval, *APOE4* apolipoprotein E ε4, *HDRS* Hamilton Depression Rating Scale

^a^Good sleep quality was taken as a reference, so aOR represents the likelihood of being Aβ positive for bad sleep quality compared with good one

### Associations between sleep experiences and AD signature neurodegeneration

Multiple logistic regression analyses revealed that sleep quality in any lifetime periods was not associated with AD-ND positivity (Table 4). However, shorter sleep duration in midlife, but not the other lifetime periods, was significantly associated with a higher AD-ND positivity rate. There were no interaction effects of midlife sleep duration and APOE4 on AD-ND positivity. These results were very similar, even after controlling for global Aβ retention as an additional covariate.

**Table 4:** Multiple logistic regression analyses with AD-ND positivity as a dependent variable

	Sleep quality				Sleep duration			
	Model 1		Model 2		Model 1		Model 2	
	aOR (95% CI)^a^	*p*	aOR (95% CI)^a^	*p*	aOR (95% CI)	*p*	aOR (95% CI)	*p*
Young adulthood	1.428 (0.426–4.786)	0.564	1.423 (0.423–4.781)	0.569	0.826 (0.628–1.088)	0.174	0.823 (0.625–1.084)	0.166
Midlife	1.989 (0.714–5.539)	0.188	1.999 (0.708–5.641)	0.191	0.679 (0.516–0.894)	0.006	0.678 (0.514–0.893)	0.006
Current	0.693 (0.236–2.036)	0.504	0.677 (0.227–2.018)	0.484	0.938 (0.745–1.182)	0.588	0.939 (0.745–1.184)	0.594

Model 1: adjusted for age, gender, education, APOE4 status, vascular risk score, use of sleep pill, Hamilton Depression Rating Score. Model 2: adjusted for covariates of model 1 + global Aβ deposition. Global Aβ deposition was natural log-transformed to achieve normal distribution

*AD-ND* Alzheimer’s disease signature region neurodegeneration, *aOR* adjusted odds ratio, *CI* confidence interval, *Aβ* beta-amyloid, *APOE4* apolipoprotein E ε4

^a^Good sleep quality was taken as a reference, so aOR represents the likelihood of being AD-ND positive for bad sleep quality compared with good one

Multiple linear regression analyses revealed that bad sleep quality in any lifetime periods was not associated with either AD-CM or AD-CT (Table 5). In terms of sleep duration, shorter duration in midlife, but not any other lifetime periods, was significantly associated with decreased AD-CM. Additional analyses did not reveal an interaction between midlife sleep duration and APOE4 and showed that sleep duration in any lifetime periods was not related to AD-CT. Similar results were obtained after further adjustment for global Aβ deposition (Additional file 1: Table S1).

**Table 5:** Multiple linear regression analyses with neurodegeneration biomarkers as dependent variables

	Sleep quality		Sleep duration	
	Beta^a^	*p*	Beta^a^	*p*
Dependent variable: AD-CM				
Young adulthood	0.011	0.880	0.135	0.054
Midlife	− 0.058	0.418	0.173	0.015
Current	− 0.013	0.871	< 0.001	0.995
Dependent variable: AD-CT				
Young adulthood	− 0.002	0.970	0.008	0.894
Midlife	− 0.017	0.779	− 0.001	0.987
Current	− 0.117	0.088	− 0.033	0.586

Adjusted for age, gender, education, apolipoprotein E ε4 status, vascular risk score, use of sleep pill, Hamilton Depression Rating Score. AD-CT were natural log-transformed to achieve normal distribution

*AD-CM* Alzheimer’s disease signature region cerebral glucose metabolism, *AD-CT* Alzheimer’s disease signature region cortical thickness

^a^Positive beta value means that better sleep quality or decreased duration of sleep is associated with decreased glucose metabolism and/or cortical thickness, respectively

## Discussion

In CN elderly individuals, bad sleep quality and shorter sleep duration during the midlife period were significantly associated with higher levels of Aβ deposition and increased AD-ND, respectively. Although current bad sleep quality appeared to be associated with increased Aβ accumulation, this relationship disappeared after controlling for the effects of midlife sleep quality. Neither sleep quality nor duration of sleep during young adulthood was related to Aβ burden or neurodegeneration. To the best of our knowledge, this study is the first to reveal the associations of sleep experience in different lifetime periods with cerebral Aβ accumulation and neurodegeneration.

Based on the temporal order of the lifetime periods, the finding that there was a significant association between bad sleep quality during the 40–50 years of age period and increased late-life Aβ deposition suggests that midlife sleep disturbances may contribute to pathological Aβ deposition. Similarly, the significant association between short sleep duration in midlife and increased AD-ND could also be interpreted as the influence of short sleep duration during midlife on the acceleration of the neurodegenerative process. Although several previous studies have reported that a greater Aβ burden is associated with poorer self-reported sleep quality in CN old adults [8–11], these studies only collected current sleep data at the time of amyloid imaging. Therefore, the results are not able to provide information regarding the direction of the relationship between sleep and Aβ deposition. Similar to previous studies, we found that current sleep quality was significantly associated with Aβ accumulation when not considering the effects of the past sleep experience. However, this difference was no longer significant when the effects of sleep quality over the preceding midlife period were considered in the same model. Taken together, the present results suggest that chronic bad sleep experiences, particularly poor sleep quality during midlife, could accelerate cerebral Aβ deposition. However, the actual influence of current sleep quality on Aβ deposition, and vice versa, remains unclear. In contrast to the results for sleep quality, there were no associations between the past or current sleep duration and Aβ accumulation in the present study, which is consistent with most previous reports [10–12, 31]. This null finding can be explained, at least in part, by the fact that an optimal duration of sleep, compared to sleep quality, will vary across the lifespan and from person to person [32].

Several studies have provided clues regarding the plausible mechanisms that may underlie the influence of sleep disturbances on cerebral Aβ accumulation. Aβ levels in the brain interstitial fluid (ISF) are directly increased by elevations in synaptic activity [33], which decreases during sleep [34]. Therefore, disturbed sleep could increase amyloid production via enhancements of synaptic activity. In terms of Aβ clearance, natural sleep increases the exchange in cerebrospinal fluid with ISF, which results in an elevated rate of Aβ clearance [35].

Regarding neurodegeneration, we found that short sleep duration during midlife was associated with decreased glucose metabolism in the brain regions that are vulnerable to AD [27, 28]; this finding remained significant even after controlling for global Aβ deposition. Evidence from experimental as well as clinical studies indicates that insufficient sleep, whether it is behavioral or disease-related, may stimulate appetite and cause disturbances in glucose metabolism [36]. Moreover, several studies have demonstrated that higher fasting serum glucose levels [37] or higher glycated hemoglobin levels [38] in non-diabetic CN older adults are associated with cerebral hypometabolism in AD signature brain regions. Furthermore, wakefulness promotes neurotoxic and oxidative stresses that are related to neurodegeneration, which increases the risk for AD [39]. Overall, the present results provide additional insights regarding the manner in which short sleep duration during the midlife period could contribute to AD-related cerebral hypometabolism via an amyloid-independent mechanism.

In contrast to the findings regarding cerebral Aβ deposition and regional hypometabolism, there were no significant associations between sleep experiences during any of the lifetime periods and cortical thickness in the AD signature regions. Only a few studies have investigated the associations between brain structures and sleep in healthy individuals. These studies have found that a reduced volume in the orbitofrontal cortex (OFC) is linked with increased early morning awakenings [40] and daytime sleepiness [41]. Furthermore, increased atrophy in the superior frontal cortex [15] and insular region [9] is associated with poor sleep quality. In the present study, additional analyses were performed assessing the previously identified brain regions, including the OFC, superior frontal cortex, and insula, but no significant associations were observed (Additional file 1: Table S2). These discrepancies may be related to the differences in subject characteristics and analysis methods among studies. Although the present study included only elderly participants, previous studies [9, 15, 40, 41] included a wide range of ages with a younger average age. Because normal aging itself influences atrophy in the prefrontal cortex, including the OFC [42], our negative results can be interpreted to mean that the association between poor sleep and brain atrophy was relatively diminished due to the aging effects in elderly individuals. Another possibility for this discrepancy could be the use of different image analysis methods; we employed an ROI-based approach whereas previous studies [9, 15, 40, 41] have used voxel-based methods.

The strengths of the present study include the evaluation of sleep experiences in different lifetime periods, the use of multimodal neuroimaging, and the recruitment of healthy elderly people with fewer depressive symptoms and few sleep disorders. However, several limitations should also be mentioned. First, we included only subjective measurements of sleep experiences and did not employ objective sleep measures. However, there is a modest relationship between self-reported sleep experiences and more objective measures using polysomnography [21, 43]. Apart from its relevance to the objectively measured sleep, self-reported sleep experiences are important because they are strongly associated with the risk for AD dementia [3] and Aβ burden [8–11], which was also observed in the present study. Furthermore, sleep questionnaires are advantageous in that they are feasible, easily used in clinical practice, and directly reflect one’s perception of their own sleep, which may incorporate their actual sleep patterns as well as the emotional response to sleep [44, 45]. Second, although all efforts were made to overcome the limitations inherent to cross-sectional studies and the lifetime sleep experiences were separately assessed over three periods of life, all data on sleep experiences were obtained based on individual recall. The recall bias of sleep assessment increases with poor cognitive function [46] and severe depressive symptom [47]. To reduce this bias as much as possible, only CN elderly subjects without depressive disorders were included in this study. As a result, the overall age-related pattern of self-reported sleep in the present subjects (Table 1) matched well with the established age-related pattern of sleep based on objective measurement, i.e., sleep quality got poorer and sleep duration became shorter as the age of the participants increased [48]. If recall bias occurs randomly, then it is likely that the relationships between sleep and the biomarkers of AD were underestimated. Therefore, our positive findings were not likely to be observed by chance. Finally, it is possible that the bad sleep experiences were related to other unmeasured etiological factors, such as poor sleep hygiene, psychological factors, and/or other subclinical medical conditions, which in and of themselves may be linked to AD processes.

## Conclusions

Our results suggest that bad sleep quality during midlife increases pathological Aβ deposition in the brain and that short sleep duration during the same period accelerates AD-related regional hypometabolism. On the other hand, the present results do not support the hypothesis that Aβ deposition or the neurodegenerative process influences sleep experiences in cognitively healthy old adults.

## Additional file

Additional file 1: **Table S1.** Multiple linear regression analyses with neurodegeneration biomarkers as dependent variable after controlling for covariates including Aβ burden, **Table S2.** Multiple linear regression analyses with cortical thickness of other than AD-signature regions as dependent variables and list of co-investigators (KBASE research group). (DOCX 35 kb)

## Data Availability

The datasets that were generated and analyzed during the present study are not publicly available due to ethical considerations and privacy restrictions. Data may be obtained from the corresponding author upon approval of the Institutional Review Board of the Seoul National University Hospital, Republic of Korea.

## Abbreviations

AD: Alzheimer’s disease
AD-CM: Alzheimer’s disease signature region cerebral glucose metabolism
AD-CT: Alzheimer’s disease signature region cortical thickness
AD-ND: Alzheimer’s disease signature neurodegeneration
APOE: Apolipoprotein E
APOE4: Apolipoprotein E ε4
Aβ: Amyloid-beta
CERAD-K: Consortium to Establish a Registry for Alzheimer’s Disease, Korean version
CN: Cognitively normal
FDG: [^18^F] Fluorodeoxyglucose
HDRS: Hamilton Depression Rating Scale
ISF: Interstitial fluid
KBASE: Korean Brain Aging Study for the Early Diagnosis and Prediction of AD
MRI: Magnetic resonance imaging
OFC: Orbitofrontal cortex
PET: Positron emission tomography
PiB: [^11^C] Pittsburgh compound B
PSQI: Pittsburgh Sleep Quality Index
ROIs: Region of interests
SUVR: Standardized uptake value ratio
VRS: Vascular risk score
