# Supplementary material for: Sleep experiences during different lifetime periods and in vivo Alzheimer pathologies
Source: Alzheimers Res Ther. 2019 Sep 12;11:79. doi: 10.1186/s13195-019-0536-6 (PMC6739958; doi:10.1186/s13195-019-0536-6)
Supplement: Supplementary file 1 — Table S1. Multiple linear regression analyses with neurodegeneration biomarkers as dependent variable after controlling for covariates including Aβ burden, Table S2. Multiple linear regression analyses with cortical thickness of other than AD-signature regions as dependent variables and list of co-investigators (KBASE research group). (DOCX 35 kb) [file 13195_2019_536_MOESM1_ESM.docx]

**Sleep experiences during different lifetime periods and in vivo Alzheimer pathologies**

**Supplemental tables**

| Table S1 Multiple linear regression analyses with neurodegeneration biomarkers as dependent variable after controlling for covariates including Aβ burden | | | | | |
| --- | --- | --- | --- | --- | --- |
|  | Sleep quality | |  | Sleep duration | |
|  | Beta^a^ | p |  | Beta^a^ | p |
|  |  |  |  |  |  |
| Dependent variable: AD-CM | | |  |  |  |
| Young adulthood | 0.018 | 0.799 |  | 0.149 | 0.033 |
| Midlife | -0.038 | 0.604 |  | 0.181 | 0.011 |
| Current | 0.003 | 0.967 |  | -0.042 | 0.966 |
|  |  |  |  |  |  |
| Dependent variable: AD-CT | | |  |  |  |
| Young adulthood | -0.002 | 0.967 |  | 0.008 | 0.899 |
| Midlife | -0.018 | 0.766 |  | -0.001 | 0.984 |
| Current | -0.120 | 0.085 |  | -0.033 | 0.588 |
| Adjusted for age, gender, education, apolipoprotein E ε4 status, vascular risk score, use of sleep pill, Hamilton Depression Rating Score, and global Aβ deposition. Global Aβ deposition and AD-CT were natural log-transformed to achieve normal distribution. Positive standardized beta means good sleep quality and decreased duration of sleep is associated with decreased glucose metabolism and cortical thickness, respectively. Aβ beta-amyloid; AD-CM Alzheimer’s disease-signature region cerebral glucose metabolism; AD-CT: Alzheimer’s disease-signature region cortical thickness. | | | | | |

| Table S2 Multiple linear regression analyses with cortical thickness of other than AD-signature regions as dependent variables | | | | | |
| --- | --- | --- | --- | --- | --- |
|  | Sleep quality | |  | Sleep duration | |
|  | Beta^a^ | p |  | Beta^a^ | p |
|  |  |  |  |  |  |
| Dependent variable: medial orbitofrontal cortex | | |  |  |  |
| Young adulthood | -0.020 | 0.773 |  | -0.090 | 0.197 |
| Midlife | -0.041 | 0.569 |  | 0.032 | 0.652 |
| Current | <0.001 | 0.998 |  | 0.099 | 0.163 |
|  |  |  |  |  |  |
| Dependent variable: superior frontal cortex | | |  |  |  |
| Young adulthood | 0.057 | 0.365 |  | -0.034 | 0.596 |
| Midlife | -0.031 | 0.627 |  | 0.022 | 0.733 |
| Current | 0.031 | 0.672 |  | 0.070 | 0.276 |
|  |  |  |  |  |  |
| Dependent variable: insula | | |  |  |  |
| Young adulthood | -0.046 | 0.506 |  | -0.068 | 0.325 |
| Midlife | -0.064 | 0.364 |  | 0.053 | 0.452 |
| Current | -0.063 | 0.432 |  | 0.012 | 0.867 |
| Adjusted for age, gender, education, apolipoprotein E ε4 status, vascular risk score, use of sleep pill, Hamilton Depression Rating Score. ^a^Positive beta value means that better sleep quality or decreased duration of sleep is associated with decreased cortical thickness. | | | | | |

**Co-investigators**

KBASE Research Group

| Name | Location | Role | Contribution |
| --- | --- | --- | --- |
| Dong Young Lee, MD, PhD | Seoul National University College of Medicine | Principal Investigator | Designed and conceptualized the cohort study; Led and supervised the cohort study; coordinated communication among study cores and study sites; acquired funding |
| Min Soo Byun, MD, PhD | Medical Research Center Seoul National University | Core PI Clinical & Executive | Supervised and coordinated the Clinical and Executive core of the cohort study |
| Dahyun Yi, PhD | Medical Research Center Seoul National University | Core PI Neuropsychology | Supervised and coordinated the Neuropsychological Core of the cohort study |
| Yu Kyeong Kim, MD, PhD | SMG-SNU Boramae Medical Center | Core PI PET | Supervised and coordinated the PET Core of the cohort study |
| Chul-Ho Sohn, MD, PhD | Seoul National University College of Medicine | Core PI MRI | Supervised and coordinated the MRI Core of the study |
| Inhee Mook-Jung, PhD | Seoul National University College of Medicine | Core PI Biomarker | Supervised and coordinated the Biomarker Core of the study |
| Murim Choi, PhD | Seoul National University | Core PI Genetics | Supervised and coordinated the Genetic Core of the study |
| Yu Jin Lee, MD, PhD | Seoul National University College of Medicine | Core PI Sleep | Supervised and coordinated the Sleep Core of the study |
| Seokyung Hahn, PhD | Seoul National University College of Medicine | Core PI Biostatistics | Supervised and coordinated the Biostatistics Core of the study |
| Hyun Jung Kim, MD | Changsan Convalescent Hospital | co-investigator | Performed clinical assessment of participants and quality control of the clinical data |
| Mun Young Chang, MD | Chung-Ang University College of Medicine | co-investigator | Coordinated an add-on study of the main cohort study |
| Seung Hoon Lee, MD | Daerim St. Mary's Hospital | co-investigator | Performed clinical assessment of participants and quality control of the clinical data |
| Na Young Han, MD | Dongrae Medical Center | co-investigator | Performed clinical assessment of participants and quality control of the clinical data |
| Jisoo Pae, MD, PhD | Genome & Company | co-investigator | Coordinated an add-on study of the main cohort study |
| Hansoo Park, MD, PhD | Genome & Company | co-investigator | Coordinated an add-on study of the main cohort study |
| Jee Wook Kim, MD, PhD | Hallym University Dongtan Sacred Heart Hospital | co-investigator | Coordinated a study site and performed participants recruitment and quality control of the clinical data |
| Young Min Choe, MD | Hallym University Dongtan Sacred Heart Hospital | co-investigator | Performed recruitment and clinical assessment of participants and monitoring of the clinical data |
| Jong-Min Lee, PhD | Hanyang University | co-investigator | Coordinated an add-on study of the main cohort study |
| Dong Woo Lee, MD, PhD | Inje University Snaggye Paik Hospital | co-investigator | Coordinated a study site and recruited participants of the cohort study |
| Bo Kyung Sohn, MD | Inje University Snaggye Paik Hospital | co-investigator | Coordinated a study site and recruited participants of the cohort study, performed clinical data analysis |
| Seok Woo Moon, MD, PhD | Konkuk University Chungju Hospital | co-investigator | Coordinated a study site and performed clinical data analysis |
| Seung-Ho Ryu, MD, PhD | Konkuk University Medical Center | co-investigator | Coordinated a study site and recruited participants |
| Man Ho Choi, PhD | Korea Institute of Science and Technology | co-investigator | Supervised and coordinated the MRI Core of the study |
| Hyewon Baek, MD | Kyunggi Provincial Hospital for the Elderly | co-investigator | Performed clinical assessment of participants and quality control of the clinical data |
| Yoon-Keun Kim, MD, PhD | MD Healthcare Inc. | co-investigator | Coordinated an add-on study of the main cohort study |
| Kang Ko, MD | National Center for Mental Health | co-investigator | Performed clinical assessment of participants and quality control of the clinical data |
| Jong-Won Kim, MD, PhD | Samsung Medical Center | co-investigator | Supervised and performed genetic analysis |
| Shin Gyeom Kim, MD, PhD | Soonchunhyang University Hospital Bucheon | co-investigator | Coordinated a study site and performed clinical data analysis |
| Sun-Ho Han, PhD | Seoul National University | co-investigator | Coordinated blood sample repository, performed blood-biomarker-related analysis |
| Joo-Youn Cho, PhD | Seoul National University | co-investigator | Coordinated and performed blood-biomarker-related analysis |
| Jae Sung Lee, PhD | Seoul National University | co-investigator | Coordinated and performed PET image data-related analysis |
| Yun-Sang Lee, PhD | Seoul National University | co-investigator | Coordinated the acquisition of the PET data and related logistics |
| Jong Inn Woo, MD, PhD | Seoul National University | co-investigator | Supervised and advised the cohort study |
| Sang Eun Kim, MD, PhD | Seoul National University Bundang Hospital | co-investigator | Coordinated the production of PET radiotracer |
| Byung Chul Lee, PhD | Seoul National University Bundang Hospital | co-investigator | Coordinated the production of PET radiotracer |
| Gi Jeong Cheon, MD, PhD | Seoul National University Hospital | co-investigator | Coordinated the acquisition of the PET data |
| Koung Mi Kang, MD | Seoul National University Hospital | co-investigator | Participated in the acquisition and clinical interpretation of the MRI/MRA data |
| Jee-Eun Park, MD, PhD | Seoul National University Hospital | co-investigator | Performed clinical and sleep-related data analysis |
| Hyeong Gon Yu, MD, PhD | Seoul National University Hospital | co-investigator | Coordinated an add-on study of the main cohort study |
| Jun-Young Lee, MD, PhD | SMG-SNU Boramae Medical Center | co-investigator | Coordinated a study site and performed participants recruitment |
| Hyo Jung Choi, MD |  | co-investigator | Performed clinical assessment of participants and quality control of the clinical data |
| Kwangsoo Kim, Ph.D | Seoul National University Hospital | co-investigator | Supervised and performed biostatistics data analysis |
| Jun Ho Lee, MD | Seoul National University Hospital | co-investigator | Coordinated participant recruitment and follow-up, performed clinical assessment of participants, quality control of the clinical data analysis |
| So Yeon Jeon, MD | Chungnam National University Hospital | co-investigator | Coordinated participant recruitment and follow-up, performed clinical assessment of participants, quality control of the clinical data |
| Sung Wook Park, MD, PhD | Seoul National University Hospital | research fellow | Performed an add-on study and data analysis |
| Woo Jin Kim, MD, PhD | Seoul National University Hospital | research fellow | Performed clinical assessment of participants and quality control of the clinical data |
| Hak Young Kim | Seoul National University Hospital | psychologist | Performed neuropsychological assessment of participants, quality control and preprocessing of the data |
| Haejung Joung | Seoul National University Hospital | psychologist | Performed neuropsychological assessment of participants. quality control and preprocessing of the data |
| Younghwa Lee | Seoul National University Hospital | psychologist | Performed neuropsychological assessment of participants, quality control and preprocessing of the data |
| Donghwi Hwang | Seoul National University | image analyst | Performed PET data analysis |
| Seung Kwan Kang | Seoul National University | image analyst | Performed PET data analysis |
| Seong A Shin | Seoul National University | image analyst | Performed PET data pre-processing |
| Jeong Yeon Hwang, MD | Seoul National University | data analyst | Performed sleep-related data analysis |
| Jong-Chan Park | Seoul National University | data analyst | Performed blood-biomarker related analysis |
| Jong-Ho Park | Samsung Medical Center | genetic data analyst | Performed genetic data analysis |
| Jieun Seo | Seoul National University | genetic data analyst | Performed genetic data analysis |
| Gi Jung Jung | Seoul National University Hospital | research coordinator | Coordinated participants recruitment, follow-up and assessment among sites, performed clinical assessment of participants and data monitoring |
| Min Jeong Kim | Seoul National University Hospital | research coordinator | Coordinated participants recruitment, Performed clinical assessment of participants |
| Han Na Lee | Seoul National University Hospital | research coordinator | Coordinated participants recruitment, follow-up and assessment among sites, performed clinical assessment of participants and data monitoring |
| Yun Jung Hwang | Seoul National University Hospital | researcher | Performed the clinical data analysis |
| Joon Hyung Jung, MD | Seoul National University Hospital | researcher | Performed the clinical data analysis |
| Kiyoung Sung, MD | Seoul National University Hospital | researcher | Performed the clinical data analysis |
| Eun Hye Kim | Seoul National University | research assistant | Coordinated and performed the collection and pre-processing of blood samples |
| Han Byul Choi | National Research Center for Dementia | administrative staff | Coordinated participant recruitment and provided administrative support |
